# Endogenous Amphiregulin promotes muscle repair and function through expansion of fibro-adipogenic progenitors during chronic *Toxoplasma gondii* infection in mice

**DOI:** 10.3389/fmicb.2026.1854938

**Published:** 2026-08-26

**Authors:** Ronzon Shihab, Andrea Alfaro-Chacon, Monica Humby, Caleb Buerger, Elizabeth A. Wohlfert

**Affiliations:** Department of Microbiology and Immunology, Jacobs School of Medicine and Biomedical Sciences, State University of New York at Buffalo, Buffalo, NY, United States

**Keywords:** Amphiregulin, fibro- adipogenic progenitor, immune response, inflammation, pathology, *Toxoplasma gondii*

## Abstract

Skeletal muscle regeneration relies on coordinated interactions between immune cells and resident stem cell populations. While most studies have focused on sterile injury, the impact of infections on muscle repair remains less understood. *Toxoplasma gondii*, a widespread parasite that establishes chronic infection in skeletal muscle and also the central nervous system, induces myositis, fibrosis, and loss of muscle function. In mice, a natural intermediate host, chronic infection sustains a robust Th1 response dominated by IFNγ-producing CD4^+^ and CD8^+^ T cells. Regulatory T cells (Tregs), which normally promote resolution of inflammation, instead adopt a pathogenic phenotype, increasing inflammation and impairing repair. Amphiregulin (Areg), a ligand of the epidermal growth factor receptor, has been implicated in tissue repair by enhancing Treg function, promoting macrophage polarization, and supporting mesenchymal differentiation. While exogenous Areg improves muscle function during chronic *T. gondii* infection, the role of endogenous Areg remains unknown. Here, we used Areg-deficient mice to investigate its contribution to muscle repair during chronic infection. We show that Areg deficiency impaired Areg deficiency impaired fibro-adipogenic progenitor (FAP) expansion, reduced IFNγ-producing CD8^+^ T cells, and diminished the frequency of Tbet^+^ Tregs. Additionally, following CTX injury, Areg-deficient mice, particularly females, exhibited impaired regeneration characterized by smaller, more heterogeneous myofibers, and increased damaged area. These findings reveal a critical role for endogenous Areg in timely FAP expansion, proper effector and regulatory T cell polarization, and efficient muscle regeneration in the setting of chronic *T. gondii* infection.

## 1 Introduction

Skeletal muscle is essential for health, supporting locomotion, metabolism, and respiration (Howard et al., 2020). The remarkable regenerative capacity of skeletal muscle relies on tightly coordinated interactions between immune cells and resident stem cell populations (Huard et al., 2002). Most studies of muscle repair have focused on sterile injury models or genetic myopathies, leaving the impact of infections on muscle regeneration comparatively understudied. Several pathogens, including *Trypanosoma cruzi* (Weaver et al., 2019), influenza virus (Desdouits et al., 2013), and *Toxoplasma gondii* (Swierzy et al., 2014), infect and persist in skeletal muscle, altering tissue homeostasis.

*T. gondii* is an obligate intracellular parasite capable of infecting any nucleated cell of warm-blooded animals (Furtado et al., 2011). By differentiating from its rapidly replicating tachyzoite form into dormant bradyzoite cysts, it establishes chronic infection within skeletal muscle fibers (Skariah et al., 2010). It is estimated that over one third of the world’s population is infected with this parasite (Wei et al., 2015). While most infections are asymptomatic, *T. gondii* has been linked to skeletal muscle pathology, including polymyositis and fibrosis, in both immunocompetent and immunocompromised individuals (Chandar et al., 1968; Gherardi et al., 1992; Hassene et al., 2008; Montoya et al., 1997). Experimental infection in mice, a natural intermediate host, recapitulates these features, with cysts and lesions accumulating in muscle fibers and impairing tissue integrity (Henry and Beverley, 1969). Because mice, like humans, are natural intermediate hosts for *T. gondii*, they provide a relevant model to study mechanisms of infection-driven muscle damage and repair.

Skeletal muscle repair is a complex process that relies on acute inflammation (Tidball, 2017). T cells and macrophages play essential roles in muscle regeneration and tissue repair (Tidball and Villalta, 2010). During the early phase, inflammatory macrophages secrete inflammatory cytokines that promote debris clearance and stimulate stem cells (Huard et al., 2002; Tidball, 2017). As repair progresses, regulatory T cells (Tregs) promote the transition of inflammatory macrophages into a restorative phenotype (Arnold et al., 2007; Burzyn et al., 2013; Pillon et al., 2013). Tregs facilitate the resolution of inflammation and activation of muscle satellite cells (MuSCs) (Burzyn et al., 2013; Pillon et al., 2013). MuSCs are the resident stem cells of skeletal muscle responsible for repairing damaged fibers through proliferation and differentiation. Alongside MuSCs, fibro-adipogenic progenitors (FAPs), a population of mesenchymal stromal cells, provide crucial support for regeneration by producing extracellular matrix components, such as collagen, laminin, and fibronectin, and by creating an environment that promotes MuSC expansion and differentiation (Uezumi et al., 2011; Uezumi et al., 2010; Molina et al., 2021). This coordinated process is essential for successful muscle repair.

In the context of *T. gondii* infection, the muscle repair program is profoundly disrupted, leading to myositis, fibrosis, and loss of muscle function (Jin et al., 2017; Melchor et al., 2020; Hatter et al., 2018). Chronically *T. gondii*-infected muscle sustains a robust Th1 response characterized by IFNγ-producing CD4^+^ and CD8^+^ T cells (Araujo, 1991; Combe et al., 2005; Gazzinelli et al., 1992; Suzuki et al., 1988). Critically, Tregs, normally central to the transition toward tissue repair, adopt a pathogenic phenotype marked by Tbet expression, thereby amplifying inflammation rather than resolving it. This pathogenic reprogramming promotes accumulation of inflammatory macrophages that fail to shift to a restorative phenotype, ultimately impairing the normal regenerative capacity of skeletal muscle (Melchor et al., 2020; Zaiss et al., 2013).

Amphiregulin (Areg), a ligand for the epidermal growth factor receptor (EGFR), has emerged as a key mediator of wound repair in diverse tissues including lung, liver, gut, and muscle (Zaiss et al., 2013; Minutti et al., 2019; Jamieson et al., 2013; Berasain and Avila, 2014; Villalta et al., 2014; Jin et al., 2018b). Produced by macrophages, innate lymphoid cells, Th2 cells, and Tregs, Areg enhances Treg suppressive function, promotes macrophage transition to restorative states, and facilitates mesenchymal differentiation (Burzyn et al., 2013; Jin et al., 2017; Zaiss et al., 2013; Kaiser et al., 2023). Our previous work showed that exogenous Areg administration in chronically *T. gondii*-infected mice reduced Tbet expression in Tregs, promoted macrophage polarization toward a restorative phenotype, and improved muscle function without compromising parasite control (Jin et al., 2018b). Yet, the role of endogenous Areg during chronic infection remains unexplored.

In this study, we used Areg-deficient mice to determine the contribution of endogenous Areg to muscle repair during chronic *T. gondii* infection (Luetteke et al., 1999; Sternlicht et al., 2005; Berasain et al., 2005). We interrogated both the stem cell compartment, examining MuSC and FAP dynamics, and the immune environment, including T cell and macrophage responses. We found that Areg deficiency impaired FAP expansion, reduced IFNγ-producing CD8^+^ T cells, diminished the frequency of Tbet^+^ Tregs, and impaired regeneration following the acute injury. These findings provide new insight into how endogenous Areg regulates muscle cell interactions during the chronic infection, highlighting a previously unrecognized mechanism of tissue repair in infection-driven myopathies.

## 2 Materials and methods

### 2.1 Mice

The mouse strain used for this research project, B6;129-*Areg^tm1Dle^*/Mmnc, RRID: MMRRC_011533-UNC, a global knockout for Amphiregulin, was obtained from the MMRRC at University of North Carolina at Chapel Hill, an NIH-funded strain repository, and was donated to the MMRRC by David C. Lee, Ph. D., University of Georgia (Luetteke et al., 1999; Sternlicht et al., 2005; Berasain et al., 2005). Cryo-preserved spermatozoa (011533-UNC-SPERM) were obtained from MMRRC. *In vitro* fertilization was performed at the Gene Targeting and Transgenic Resource, Roswell Park Comprehensive Cancer Center, Buffalo, NY, United States. Mice were subsequently bred in the Laboratory Animal Facility of Jacobs School of Medicine and Biomedical Sciences. All procedures involving mice were reviewed and approved by the Institutional Animal Care and Use Committee at the University at Buffalo (MIC14103).

### 2.2 Infection

RFP-expressing ME49 *T. gondii* cysts were prepared by homogenizing the brains of chronically infected mice (≥30 days post-infection) in PBS (graciously provided by M. Grigg). Cysts were counted in four 20 μL aliquots with a fluorescence microscope. Mice were orally gavaged with 4 to 10 cysts. Weights were monitored once every 2 days from D0 to D6, twice a day from D8 to D12, and once weekly thereafter.

### 2.3 Muscle injection

For acute injury, 30 days post infection (dpi) mice were anesthetized and injected in quadriceps, gastrocnemius, and tibialis anterior of the right hind limb with 30 μL at 0.03 mg/mL of CTX (Accurate Chemical and Scientific Corp.).

### 2.4 Isolating lymphocytes, satellite cells, and fibro-adipogenic progenitors from tissues

Lymphocytes from muscle: Mice were euthanized with CO_2_ and perfused with PBS (pH 7.2). Quadriceps, gastrocnemius, and tibialis anterior were harvested, minced, and digested (RPMI 1640, 1% penicillin–streptomycin [Gibco], 2 mM L-glutamine [Corning], 2 mg/mL Collagenase Type II [Gibco], 0.5 mg/mL DNase [Sigma-Aldrich], 25 mM HEPES [Corning], 55 μM DMSO [Gibco]) for 30 min at 37 °C. After passing through 70 μm filter, the lymphocytes were then purified using a 37.5% Percoll gradient [GE Healthcare] and resuspended in 10% media (RPMI 1640, 1% penicillin–streptomycin [Gibco], 10% FBS, 2 mM L-glutamine [Corning], 25 mM HEPES [Corning], 55 μM DMSO [Gibco]) as single cell suspension.

Lymphocytes from spleen: After harvesting, spleen were crushed through a 70 μm filter. Red blood cells were lysed using ACK Lysing Buffer (Gibco) for 2 min and resuspended in 10% media as single cell suspension.

Satellite cells and fibro-adipogenic progenitors from muscle: After harvesting and mincing quadriceps, gastrocnemius, and tibialis anterior, the tissues are digested (RPMI 1640, 1% penicillin–streptomycin [Gibco], 2 nM L-glutamine [Corning], 800 U/mL Collagenase Type II [Gibco], 25 mM HEPES [Corning], 55 μM DMSO [Gibco]) for 1 h in 37 °C water bath. The tissue suspension was then spun down and media replaced with 1,000 U/mL Collagenase Type II and 11 U/mL Dispase (Gibco) in PBS and digested for 30 min in 37 °C water bath. The cell suspension was then passed through a 20-gauge needle, filtered through a 40 μm filter and resuspended in 10% media as single cell suspension.

### 2.5 Flow cytometric analysis

In HBSS, single cell suspensions were stained with Live/Dead Fixable Blue Dead Cell Stain (Thermo Fisher Scientific) and extracellular antibodies in HBSS. Cells were fixed and permeabilized (Intracellular Fixation and Permeabilization Buffer Set, eBioscience), then washed and stained with intracellular antibodies in Permeabilization Buffer (eBioscience). For biotinylated antibodies, streptavidin staining was also done using the Permeabilization Buffer. Samples were washed and resuspended in FACS buffer (PBS, 2% FBS, 2 mM EDTA, 0.0001% sodium azide). CountBright Absolute Counting Beads (Life Technologies) were used to calculate absolute cell numbers.

### 2.6 Antibodies

Antibodies used for our assays: anti-TCRß-APC-Cy7 (clone H57-597; BD Biosciences), anti-CD4-PE-Cy7 (clone RM4-5; BD Biosciences), anti-CD8ß-PerCP-Cy5.5 (clone YTS1567.7; Biolegend), anti-FoxP3-FITC (clone FJK-16 s; Invitrogen), anti-Tbet-eFlour660 (clone O4-46; BD Biosciences). Anti-Ki67-AF700 (clone B56; BD Biosciences), anti-IFNγ-PE (clone XMG1.2; Invitrogen), anti-CD45-APC-eFlour780 (clone 30-F11; Invitrogen), anti-CD31-APC (clone MEC13.3; Biolegend), anti-Sca1-Pacific Blue (clone D7; Biolegend), anti-integrin alpha7-PE (clone 334908; R&D Systems), anti-CD34-FITC (clone RAM34; Invitrogen); anti-VCAM1-PerCP-Cy5.5 (clone 429; Biolegend), anti-Tie2-Biotin (clone TEK4; Biolegend), streptavidin-PE-CF594 (BD Biosciences), anti-CD140a-BV605 (clone APA5; Biolegend), FC block (BD Biosciences).

### 2.7 Parasite burden quantification

DNA is extracted from muscle tissue using DNeasy Blood and Tissue kit (Qiagen). Parasite burden was quantified through qPCR using the *T. gondii*-specific *B1* gene (forward: 5’-TCCCCTCTGCTG GCGAAAAGT −3′, reverse: 5’ AGCGTTCGTGGTCAACTATCG ATTG-3′). Ct values were compared with a *B1* amplification standard curve of known DNA concentrations.

### 2.8 Muscle histopathology

Mice were perfused with PBS and Tibialis anterior (TA) muscles were isolated and cryopreserved and cryosectioned at 10 μm. Muscle sections were stained with wheat germ agglutinin (WGA) conjugated with Alexa Fluor 488 (1:100, Thermo Fisher Scientific) and DAPI (Sigma-Aldrich). Images were captured using a Leica DM6 B microscope, acquiring whole cross-sections at the mid-belly of the TA muscle for each animal. Image analysis was performed using FIJI (ImageJ), and all myofibers present in the entire cross-section were segmented and included in the quantification. Percentage damaged area was determined by binarization of the WGA-stained area relative to the total muscle area. Myofiber cross-sectional area (CSA) was quantified by segmentation analysis and subsequent measurement. Cross-sectional area was measured using a modified version of Open-CSAM FIJI plugin (Desgeorges et al., 2019), where the Huang thresholding method of segmentation was replaced by the MorphoLibJ Classic Watershed (Legland et al., 2016) method of segmentation.

### 2.9 Statistical analysis

All statistical analyses were performed using GraphPad Prism 10 on data obtained from biologically independent mice. For comparisons between two groups, non-parametric Mann–Whitney tests were used unless otherwise specified, and results are reported as median ± interquartile range or mean ± SEM according to figure legends. For experiments involving more than one factor, two-way ANOVA was applied followed by appropriate *post hoc* multiple-comparison tests to assess pairwise differences. Sample sizes (*n*) refer to individual animals and are indicated in each figure legend. Statistical significance was defined as *p* < *0.05*, and exact *p* values are provided where relevant in the figure legends.

## 3 Results

### 3.1 Areg deficiency does not alter MuSC dynamics during chronic *Toxoplasma gondii* infection

**Figure 1:**
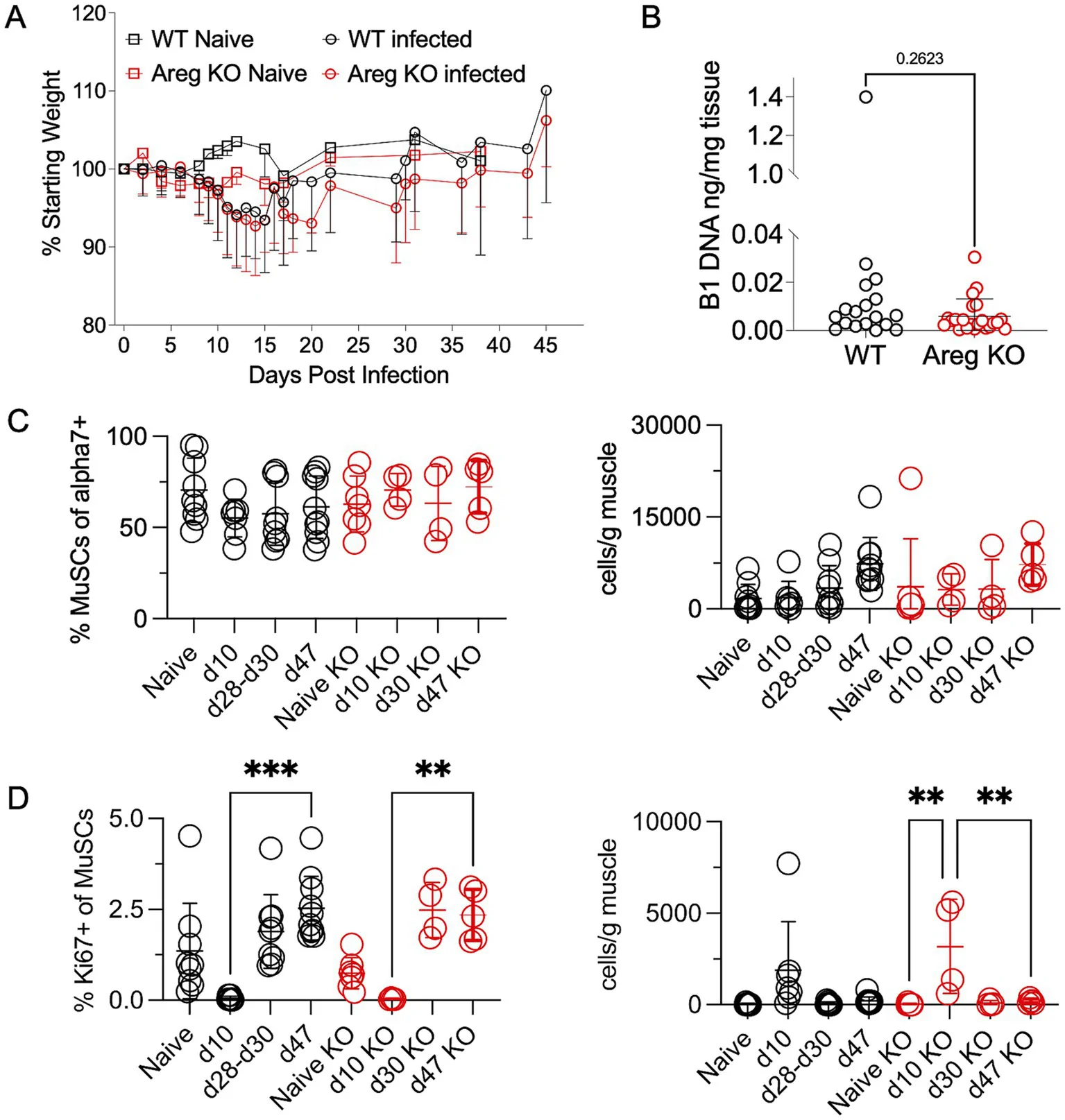
Effects of Areg deficiency on MuSCs during chronic *T. gondii* infection. **(A)** Body weight was monitored up to 47 days. **(B)** qRT-PCR quantification of parasite burden by *T. gondii* specific *B1* gene. Frequency of parent (left panel) and normalized cell counts (right panel) of **(C)** MuSCs, and **(D)** Ki67+ MuSCs populations throughout infection for WT and Areg KO mice. WT: Naïve *n* = 9, d10 *n* = 7, d28–30 *n* = 11, d47 *n* = 10; Areg KO: Naïve *n* = 7, d10 *n* = 4, d30 *n* = 3, d47 *n* = 5. Both WT and Areg KO groups contain male and female mice. Two-way ANOVA, Tukey’s multiple comparisons, * *p* < 0.05, ** *p* < 0.01, *** *p* < 0.001.

We examined *Toxoplasma* infection in Amphiregulin-deficient mice (Areg KO) to determine the role of endogenous Areg during chronic *T. gondii* infection in the skeletal muscle. Previously, we reported that *T. gondii* infection results in muscle damage and decreased muscle function in mice. Furthermore, we found that Areg supplementation rescued the regenerative response by increasing myogenic factors and improving repair and function (Jin et al., 2017; Jin et al., 2018b). Areg has been implicated in the wound repair response of skeletal muscle Tregs (Burzyn et al., 2013). Many cell types may produce Areg including Tregs, ILC2s, Th2 cells, macrophages, epithelial cells, and fibroblasts; however, Tregs are thought to be the most important source in skeletal muscle repair (Burzyn et al., 2013; Zaiss et al., 2013; Minutti et al., 2019; Berasain and Avila, 2014; Kabat et al., 2022). We found that Areg KO mice survive the acute infection and progress to chronic infection. C57BL/6 mice infected with *T. gondii* lose weight during the acute phase, and Areg KO mice lose comparable amounts of weight (Figure 1A). Starting body weights of WT and Areg KO mice in naïve and infected cohorts are shown in Supplementary Figure 1. We next quantified the parasite burden in the skeletal muscle by qPCR and found that WT and Areg KO mice have similar levels of *T. gondii* at varying cyst doses (Figure 1B).

Areg produced by skeletal muscle Tregs (SkmTregs) is important in muscle repair and thought to target satellite cells, also called muscle stem cells (MuSCs) (Burzyn et al., 2013). Therefore, we asked whether Areg deficiency impacted the MuSCs during chronic *T. gondii* infection. MuSCs were analyzed by flow cytometry. We excluded endothelial and immune cells (CD45^−^CD31^−^) and gated on integrin-alpha7^+^CD34^+^ cells, which are previously defined MuSCs surface markers (Maesner et al., 2016). We observed no differences in overall MuSC frequency across time points in either WT or Areg KO mice (Figure 1C, left panel). While MuSCs counts also show no significant differences, we observed a trend of gradual expansion in MuSCs during *T. gondii* infection (Figure 1C, right panel). The MuSC population is actively proliferating as evidenced by Ki67 expression. While the frequency of Ki67^+^ MuSCs shows a decrease at day 10 post infection and significant increases into the chronic stage of infection for both WT and Areg KO mice (Figure 1D, left panel), the cell counts show that at day 10 there is a spike in increased proliferating MuSCs which then drop to near baseline numbers (Figure 1D, right panel). Our data reflect that lack of endogenous Areg does not impact the dynamics of MuSCs during chronic *T. gondii* infection.

### 3.2 Areg deficiency delays the expansion of FAPs in skeletal muscle during chronic *Toxoplasma gondii* infection

**Figure 2:**
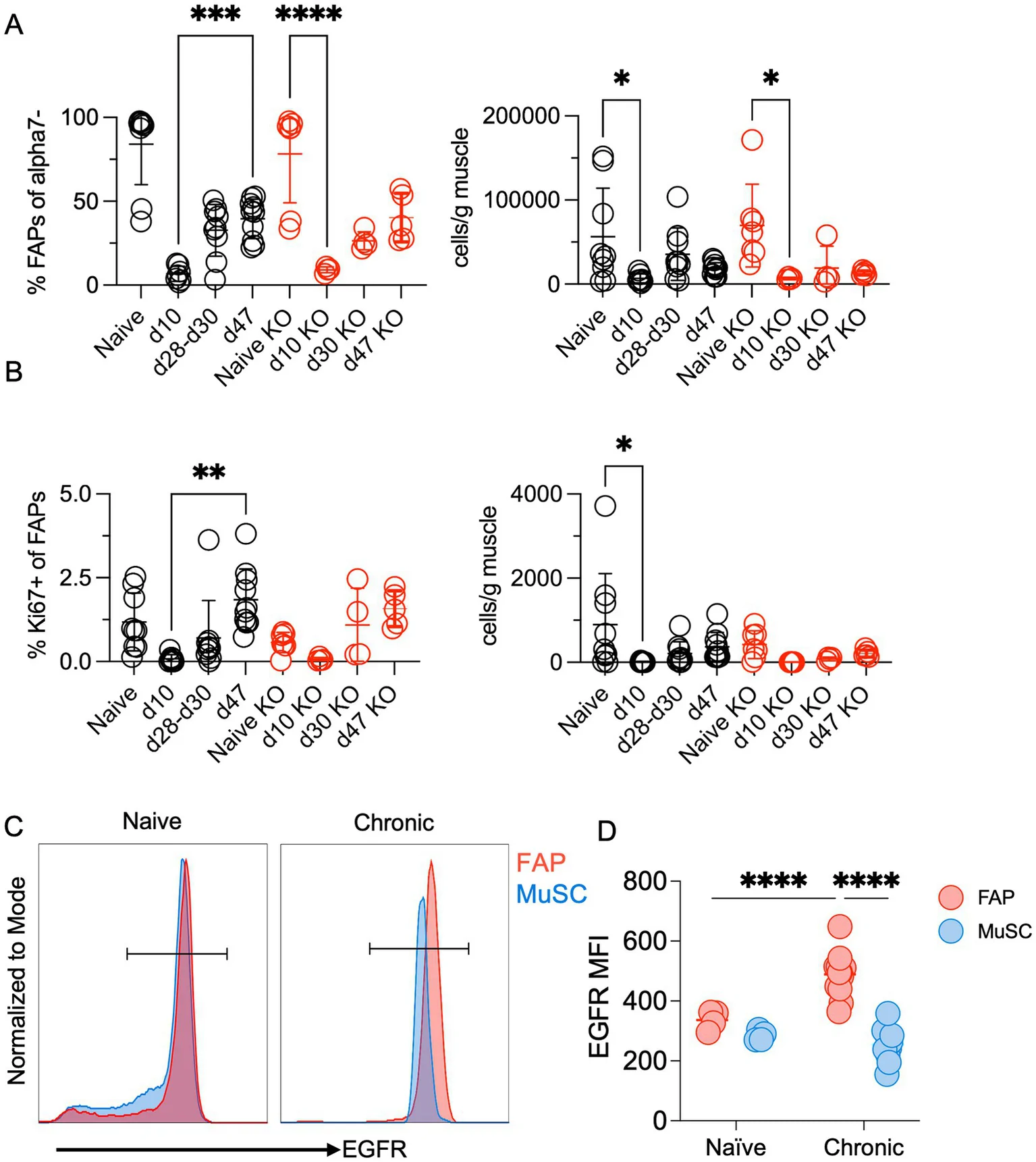
Effects of Areg deficiency on FAPs during chronic *T. gondii* infection. Frequency of parent (left panel) and normalized cell counts (right panel) of **(A)** FAPs, and **(B)** Ki67+ FAPs populations throughout infection for WT and Areg KO mice. **(C)** Representative histograms of EGFR^+^ FAPs (red) and MuSCs (blue) from WT naïve and chronically infected (d30) mice. **(D)** Quantification of EGFR mean fluorescence intensity (MFI) in FAPs and MuSCs from naïve and chronically infected mice. WT: Naïve *n* = 9, d10 *n* = 7, d28–30 *n* = 11, d47 *n* = 10; Areg KO: Naïve *n* = 7, d10 *n* = 4, d30 *n* = 3, d47 *n* = 5. Both WT and Areg KO groups contain male and female mice. Two-way ANOVA, Tukey’s multiple comparisons, * *p* < 0.05, ** *p* < 0.01, *** *p* < 0.001, **** *p* < 0.0001.

FAPs are a population of mesenchymal stromal cells that produce extracellular matrix components, creating an environment that supports muscle regeneration through MuSC expansion and differentiation (Uezumi et al., 2011; Uezumi et al., 2010; Molina et al., 2021). The complexity of FAPs has recently been revealed in response to muscle injury (Uezumi et al., 2011; Molina et al., 2021; Santini et al., 2020; Joe et al., 2010; Uapinyoying, 2023). Given the important role in muscle regeneration, we evaluated total FAPs based on expression of CD45^−^, alpha7^−^, CD31^−^ and PDGFRα^+^ (CD140α) (Uezumi et al., 2010; Santini et al., 2020; Joe et al., 2010; Uapinyoying, 2023; Petrilli et al., 2020; Yaghi et al., 2023). As shown in Figure 2A, total FAP frequency and numbers are similar during infection in control and Areg-deficient mice. To identify FAPs, we gated on live cells and excluded immune and endothelial cells (CD45^−^CD31^−^) and MuSCs (integrin-alpha7^−^). Because *T. gondii* infection has been shown to lead to muscle damage during chronic infection (Jin et al., 2017) and FAPs are highly responsive to skeletomuscular damage, we hypothesized that chronically infected WT mice would have increased FAP presence as infection progresses compared to infected Areg KO mice. When we observed FAP numbers (Figure 2A, right panel), we find in both genotypes there is a significant decrease at the acute infection time point (day 10) compared to uninfected mice.

To further investigate if Areg impacted FAPs during infection, we investigated the proliferating population of FAPs, defined by Ki67 expression (Figure 2B). FAPs are highly responsive to damage and expand rapidly (Joe et al., 2010; Petrilli et al., 2020). Because *T. gondii* has been shown to cause muscle damage and fibrosis (Melchor et al., 2020), we hypothesized that FAPs would proliferate more robustly in WT mice than in Areg KO mice. We observed a drop in the frequency of Ki67^+^ FAPs at the acute time point for both WT and Areg KO mice relative to naïve mice. As the infection progresses, WT mice show a continual increase in proliferating FAP numbers (Figure 2B, right panel), with significantly higher numbers at day 47 than day 10. While this trend in frequency is mirrored in the Areg KO mice, chronically infected KO mice do not display the significant increase in Ki67^+^ FAPs seen in WT mice. When we observed cell numbers of proliferating FAPs, WT mice show a significant decrease at the acute time point and, while no further significant changes are detected, they displayed a trend of increasing Ki67^+^ FAPs as the infection progresses. Areg KO mice do not exhibit a similar trend; by day 47, the average number of proliferating FAPs is approximately half that in WT mice. Since we observed changes in FAP responses, we asked whether FAPs express EGFR. At homeostasis, we find that FAPs and MuSCs express similar levels of EGFR (Figure 2C, left histogram). However, MuSCs do not maintain this EGFR expression during chronic infection, whereas FAPs upregulate EGFR and display significantly higher EGFR mean fluorescence intensity (MFI) during chronic infection compared to naïve conditions (Figures 2C,D). These data support the notion that Areg deficiency could alter FAP responsiveness during chronic *T. gondii* infection.

**Figure 3:**
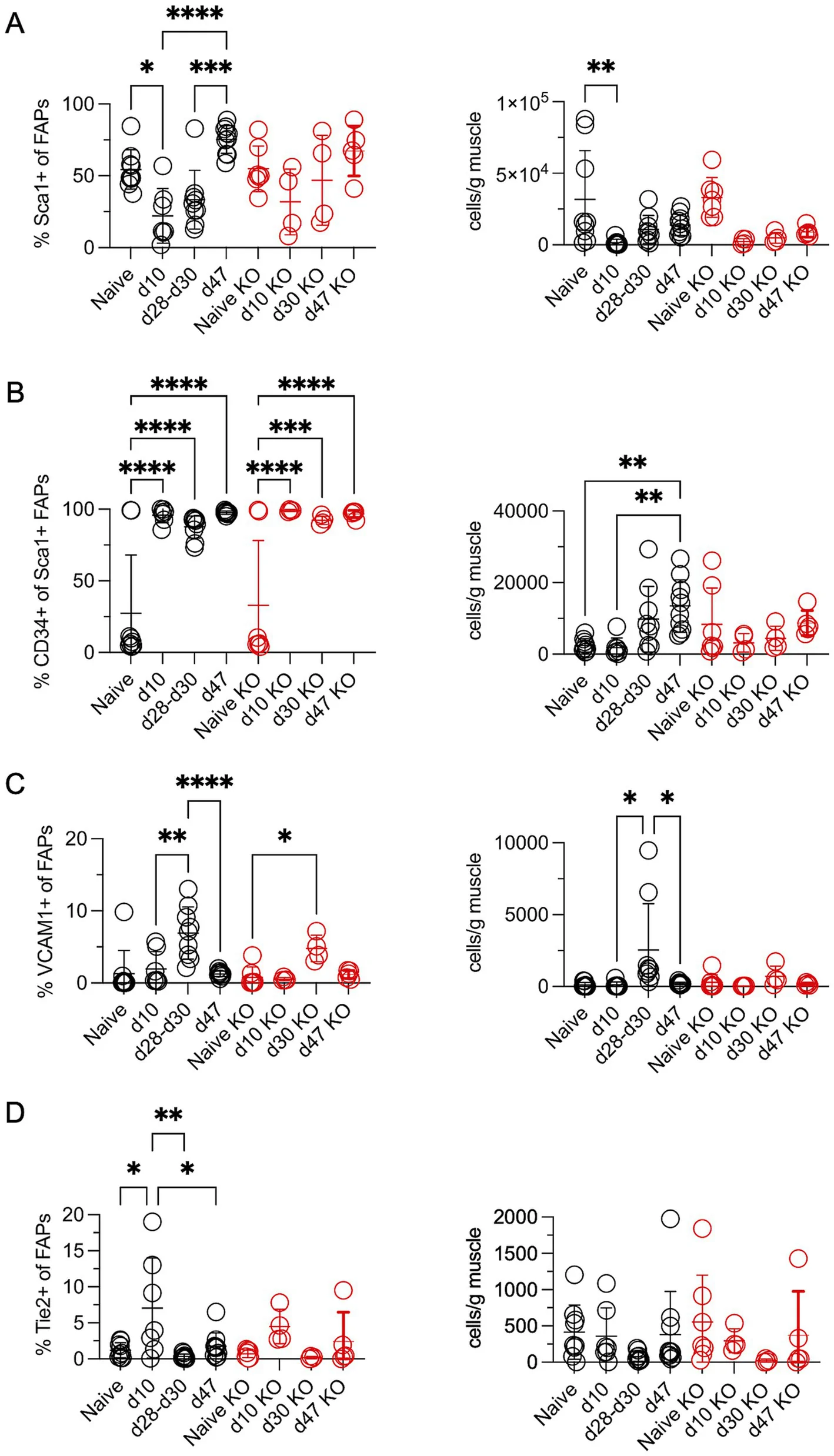
Effects of Areg deficiency on FAP activation and subpopulations during chronic *T. gondii* infection. Frequency of parent (left panel) and normalized cell counts (right panel) of **(A)** Sca1+ FAP, **(B)** CD34+ FAP, **(C)** VCAM1+ FAPs, and **(D)** Tie2+ FAP subpopulations throughout infection for WT and Areg KO mice. WT: naïve *n* = 9, d10 *n* = 7, d28–30 *n* = 11, d47 *n* = 10; Areg KO: naïve *n* = 7, d10 *n* = 4, d30 *n* = 3, d47 *n* = 5. Both WT and Areg KO groups contain male and female mice. Two-way ANOVA, Tukey’s multiple comparisons, * *p* < 0.05, ** *p* < 0.01, *** *p* < 0.001, **** *p* < 0.0001.

The heterogeneity of FAP subpopulations is still not well established. Studies using single-cell RNA sequencing have transcriptionally defined FAP subtypes in dystrophic and injury models (Uapinyoying, 2023; Yaghi et al., 2023). However, these populations have not been defined in an infection setting that leads to muscle damage. Studies in MuSCs have reported that CD34 denotes a quiescent stem cell population but also promotes MuSC entry into the cell cycle (Beauchamp et al., 2000; Alfaro et al., 2011). We examined the expression of Sca1 and CD34 on FAPs, markers of stemness, throughout infection. We observed a dynamic Sca1^+^ population during chronic *T. gondii* infection. In WT mice, Sca1^+^ FAP frequency drops significantly at the acute stage but then shows significant increase as the infection progresses (Figure 3A, left panel). Areg KO mice display a similar trend but while there is a trend for increased frequency after acute phase of infection it did not statistically significantly different from the naïve timepoint. When we examine cell counts of Sca1^+^ FAPs, WT mice show a significant drop at the acute phase compared to naïve mice and display a gradual increasing trend as infection progresses. Areg KO mice mirror this trend in Sca1^+^ FAP numbers but the changes are not significant (Figure 3A, right panel). We find that CD34 largely co-expresses with Sca1^+^ FAPs in both WT and Areg KO mice (Figure 3B, left panel). When we examine cell numbers, WT mice show a gradual and significant increase in CD34^+^Sca1^+^ FAPs as infection progresses (Figure 3B, right panel). Areg KO mice also show similar changes in the cell number but these changes are not statistically significant compared to the naïve timepoint or the WT control.

We were also able to observe by flow cytometry two other subsets of FAPs that have been previously defined, characterized by expression of VCAM1 and Tie2 (Malecova et al., 2018). VCAM1^+^ FAPs have been reported as a more activated state with a pro-fibrotic gene expression profile and are found almost exclusively in injured muscles (both in acute and dystrophic conditions). Tie2 has been reported as being predominantly expressed in uninjured muscles, defining a quiescent subpopulation of FAPs. The dynamics of these two subpopulations have never been studied in an infection setting that also leads to muscle damage, such as *T. gondii* infection. We also wanted to ask whether endogenous Areg affects these dynamics during chronic infection. We observe that the frequency of VCAM1^+^ FAPs in WT mice significantly increases and peaks around day 30 of infection, reaching a mean of 6% of all FAPs, and then shows a sharp decrease at day 47 (Figure 3C, left and right panel). Areg KO mice display a similar increase of VCAM1^+^ frequency at day 30 and a decreasing trend at day 47. When we examine cell counts of VCAM1^+^ FAPs, WT mice have significantly higher numbers at day 30 while Areg KO mice did not display any significant changes during infection. When we observe the Tie2^+^ FAP population, frequencies peak at day 10 of infection for both WT and KO mice; however, only the WT mice show a significant difference compared to naïve controls (Figure 3D, left panel). When we examine cell counts, there are no significant differences for either WT or Areg KO mice, but there is a trend toward decreasing Tie2^+^ FAPs numbers, reaching minimal numbers at day 30 (Figure 3D, right panel).

### 3.3 Areg deficiency has no impact on inflammatory and restorative macrophages during chronic *Toxoplasma gondii* infection

**Figure 4:**
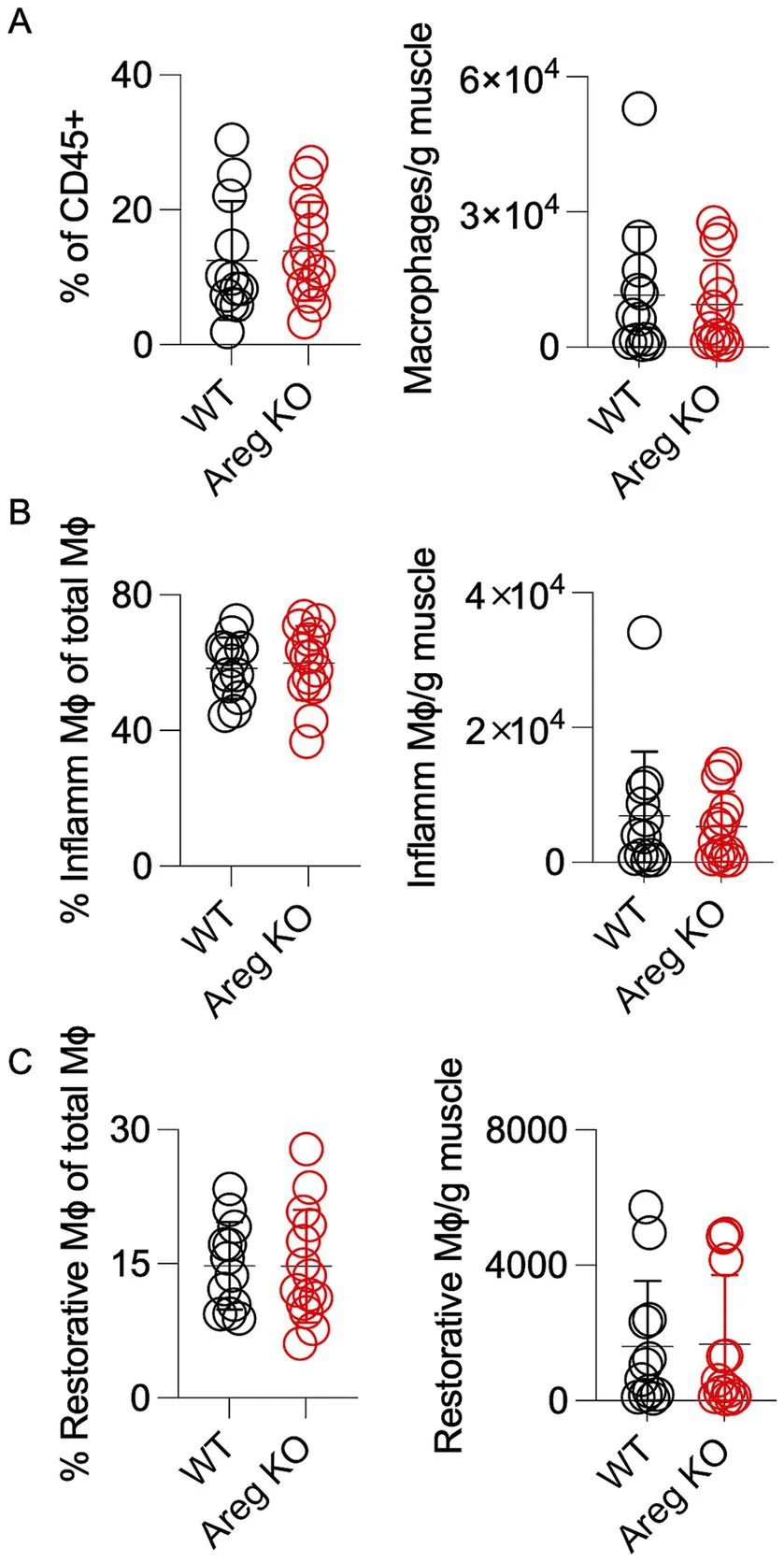
Areg deficiency does not alter monocyte or macrophage populations in the skeletal muscle during *T. gondii* infection. Frequency of parent (left panel) and normalized cell counts (right panel) of **(A)** total macrophage population, **(B)** inflammatory, and **(C)** restorative subpopulations from 30 days post infection in WT or Areg KO mice. WT and Areg KO groups contain male and female mice. WT *n* = 12, Areg KO *n* = 14. Mann Whitney test, * *p* < 0.05, ** *p* < 0.01, *** *p* < 0.001.

Muscle repair requires a tightly regulated immune response in addition to the activity of FAPs and MuSCs. Macrophage polarization is crucial for muscle repair. The inflammatory phenotype is necessary for both anti-parasitic killing mechanisms and for clearing cellular debris (Arnold et al., 2007; Park and Hunter, 2020). However, macrophages need to transition to a restorative phenotype to promote muscle repair. We previously reported that during chronic *T. gondii* infection, although there is a transition to restorative macrophages, there is a persistence in the inflammatory macrophage population that hinders muscle regeneration (Jin et al., 2017). Upon exogenous Areg administration, we reported an increased restorative macrophage population and better repair (Jin et al., 2018b). We then asked what role endogenous Areg plays in macrophage polarization during chronic *T. gondii* infection. At day 30 pi, we analyzed the total macrophages (Figure 4A, left and right panel), inflammatory (Ly6C^+^) macrophages (Figure 4B, left and right panel) and restorative (CD206^+^) macrophages (Figure 4C, left and right panel). We find no differences in the frequency or absolute number of these populations between genotypes during chronic *T. gondii* infection. Gating strategy for muscle macrophages is shown in Supplementary Figure 2.

### 3.4 Areg KO mice have decreased inflammatory CD8^+^ T cells during chronic *Toxoplasma gondii* infection

**Figure 5:**
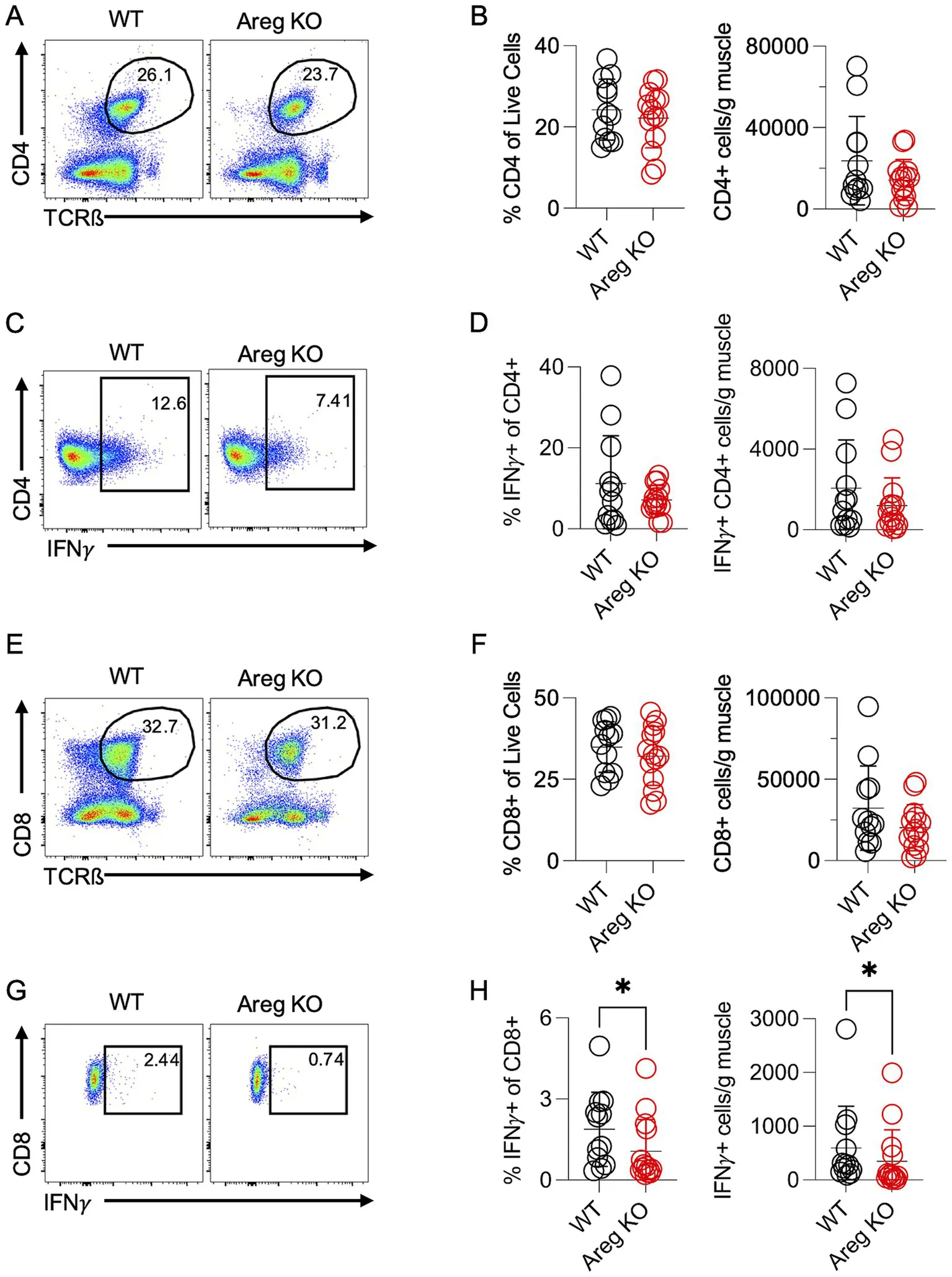
Effects of Areg deficiency on t cell populations. **(A,C,E,G)** Show representative flow plots of t cell populations from chronically infected WT or Areg KO mice. Frequency of parent (left panel) and normalized cell counts (right panel) of **(B)** CD4+ t cells **(D)** IFN𝛾+CD4 + t cells, **(F)** CD8+ t cells and **(H)** IFN𝛾+CD8+ t cells from chronically infected WT or Areg KO mice. WT *n* = 12, Areg KO *n* = 14. Both WT and Areg KO groups contain male and female mice. Mann Whitney test, * *p* < 0.05, ** *p* < 0.01, *** *p* < 0.001.

*T. gondii* induces a Th-1 inflammatory response resulting in the production of IFN𝛾, which is crucial for parasite control (Suzuki et al., 1989; Suzuki et al., 1990; Lüder, 2024). Previous studies demonstrated that exogenous Areg administration promotes wound repair without affecting the immune response or pathogen burden in various infection or toxic settings (Jamieson et al., 2013; Jin et al., 2018b; Arpaia et al., 2015; Monticelli et al., 2011). We wanted to ask whether endogenous Areg influenced the protective immune response during chronic *T. gondii* infection. We chose the day 30 time point as our previous reports demonstrated increased CD4^+^ and CD8^+^ T cell presence in the muscle. At day 30, we observe no differences between WT and Areg KO mice in both CD4^+^ and CD8^+^ T cell frequencies and cell counts (Figures 5A,B,E,F). We then investigated the inflammatory compartment by assaying for the presence of IFN𝛾-producing CD4^+^ and CD8^+^ T cells. IFN𝛾^+^CD4^+^ T cells do not exhibit significant differences in frequency or cell counts between WT and Areg KO mice (Figures 5C,D). Interestingly, however, we find that Areg KO mice have significantly fewer IFN𝛾^+^CD8^+^ T cells during chronic infection (Figures 5G,H).

### 3.5 Areg deficient mice exhibit decreased Tbet+ Tregs

**Figure 6:**
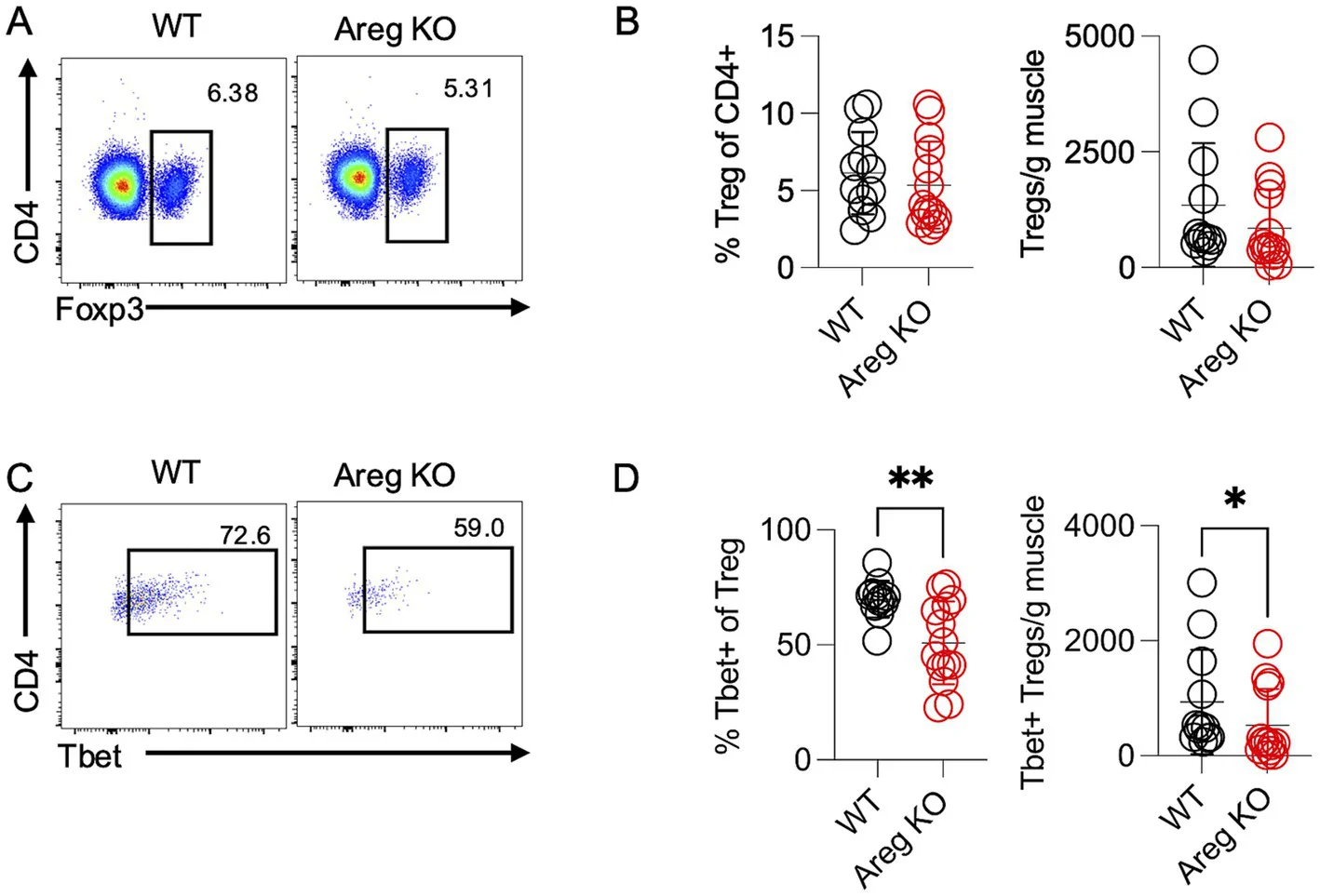
Effects of Areg deficiency on Tregs. **(A,C)** Show representative flow plots of Tregs (CD4+ FoxP3+) and Tbet+ Tregs, respectively. Frequency of parent (left panel) and normalized cell counts (right panel) of **(B)** Tregs and **(D)** Tbet+ Tregs from chronically infected WT or Areg KO mice. WT *n* = 12, Areg KO *n* = 14. Both WT and Areg KO groups contain male and female mice. Mann Whitney test, * *p* < 0.05, ** *p* < 0.01, *** *p* < 0.001.

Tregs play a crucial role in the muscle repair process. In acute injury settings, Tregs have been reported as being integral to the transition from an inflammatory to a restorative macrophages phenotype, in addition to being an important source of Areg (Burzyn et al., 2013; Arpaia et al., 2015). When we reported that inflammatory macrophages were accumulating during chronic infection, we observed the emergence of Th1-like Tregs with increased Tbet expression (Jin et al., 2017). Areg treatment, however, decreased Tbet^+^ Tregs which coincided with increased restorative macrophages and improved muscle function (Park and Hunter, 2020). Furthermore, it has also been reported that Areg enhances Treg suppressive function (Zaiss et al., 2013). We wanted to interrogate what role endogenous Areg plays in the Treg population during *T. gondii* infection. We analyzed Tregs by flow cytometry. We find no difference in total Tregs during chronic infection between WT and Areg KO mice (Figures 6A,B). However, we find that Areg KO mice have significantly fewer Tbet^+^ Tregs (Figures 6C,D).

### 3.6 Areg deficiency impairs skeletal muscle regeneration following CTX injury in chronically infected mice

Thus far, we have observed in FAP and Treg populations, both cell types that are important in muscle repair. We also observed lower levels of IFNγ, a cytokine known to interfere with muscle regeneration. Therefore, we hypothesized that these alterations may compromise regenerative capacity following acute injury. To test this, we used our previously established regeneration model in the setting of chronic infection (Jin et al., 2018a). Mice were infected with *T. gondii* and, at 30 dpi, were intramuscularly administered cardiotoxin (CTX) to induce a synchronous acute muscle injury. Parameters of muscle regeneration were evaluated 10 days post-injury (40 dpi) in wild type and Areg KO mice.

**Figure 7:**
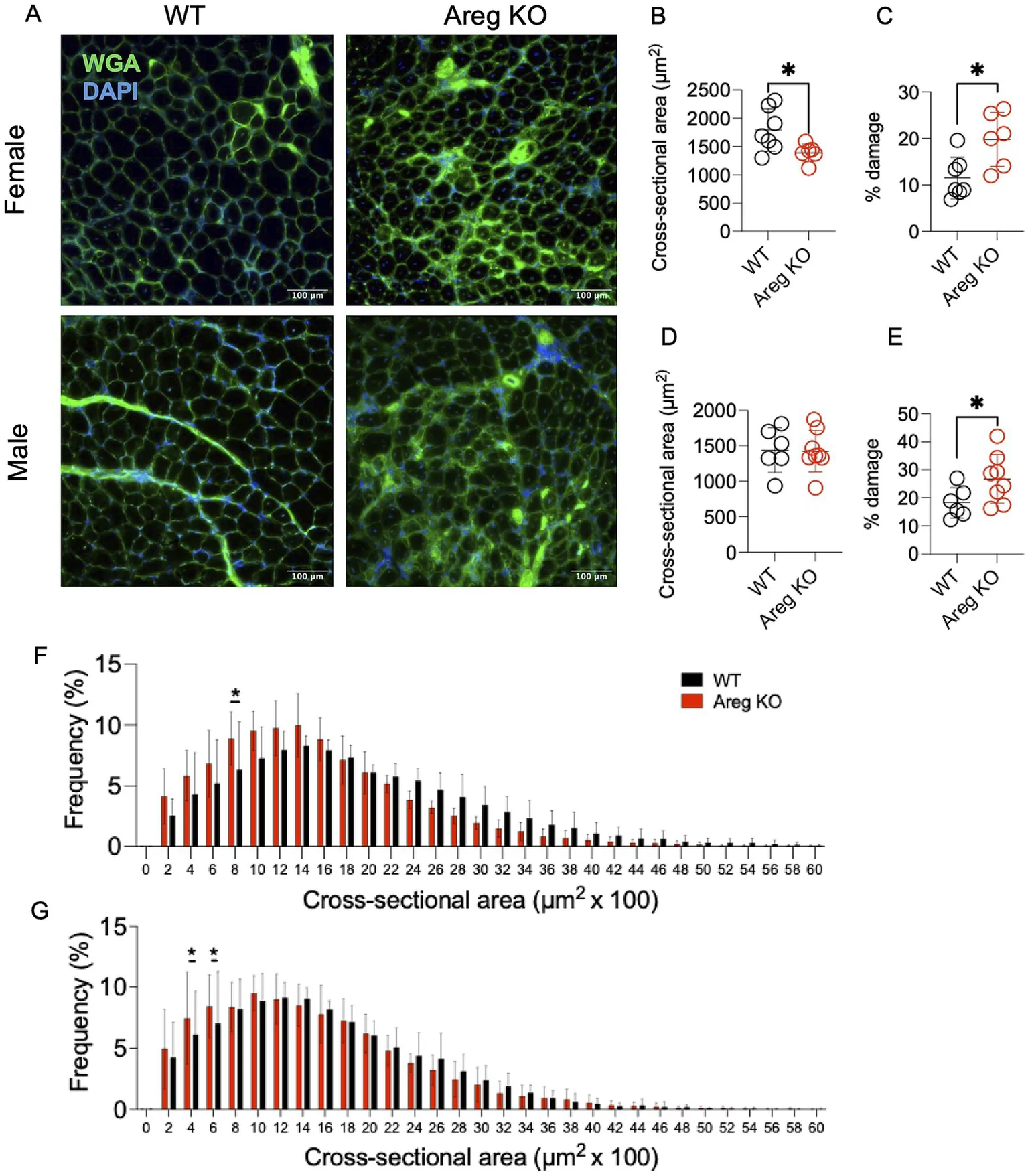
Effects of Areg deficiency on skeletal muscle morphology 10 days post-CTX injury. **(A)** Show representative images of skeletal muscle cross-sections from Areg WT and Areg KO mice 10 days after CTX injury. Female (top) and male (bottom) mice are shown. Nuclei were stained with DAPI (blue), and sarcolemma was labeled with WGA (green). Myofiber cross-sectional area and percentage of damaged area in **(B,C)** females and **(D,E)** males. Frequency distribution of myofiber cross-sectional area in **(F)** females and **(G)** males. WT (*n* = 6–7) and Areg KO (*n* = 6–7). Mann–Whitney, and two-way ANOVA, * *p* < 0.05, ** *p* < 0.01, *** *p* < 0.001.

We observed that both female and male Areg KO mice exhibit smaller, more rounded and more heterogeneous myofibers, with areas consistent with delayed progression of repair (Figure 7A). Quantification of myofiber cross-sectional area (CSA) shows a significant reduction in CSA in Areg KO females relative to WT controls (Figure 7B) but no difference in male KO (Figure 7D). Consistent with impaired regeneration, both Areg KO females and KO males display a significantly higher percentage of damaged area compared to WT mice (Figures 7C,E). Analysis of CSA frequency distribution further reveals a leftward shift in both Areg KO females and KO males, indicating an increased proportion of smaller fibers and a reduction in larger regenerated fibers (Figures 7F,G). Together with the delayed FAP expansion observed in Areg-deficient mice, these findings indicate that endogenous Areg is required to support efficient muscle regeneration in the context of chronic *T. gondii* infection.

## 4 Discussion

Areg has been studied in various damage and infection settings in tissue such as the muscle, lung, and gut. The role of Areg is highly context dependent. Areg may be a fibrotic factor, but also it is reported as being a pro-restorative growth factor that can be produced by various cells such as Tregs, macrophages, mast cells, eosinophils and type 2 innate lymphoid cells (Jin et al., 2017; Zaiss et al., 2013; Minutti et al., 2019; Jin et al., 2018b; Arpaia et al., 2015). Most studies administer exogenous Areg to investigate its reparative effects, including our own study in chronic infection. We asked in this study what the role of endogenous Areg was in *T. gondii*-myositis and resultant tissue damage. Our group previously reported that chronic *T. gondii* infection causes myositis in mice, with increased fatiguability and muscle tissue damage, and that Areg treatment improves muscle repair and function (Jin et al., 2018b). Because muscle repair ultimately requires the activation and proliferation of MuSCs, we hypothesized that, compared to WT mice, Areg-deficient mice would have reduced numbers of total and proliferating MuSCs. Unexpectedly, our results indicated that loss of endogenous Areg does not impair the MuSCs expansion during infection. It is possible that the effects observed in our previous report with Areg treatment were due to the abnormally higher availability of the cytokine, whereas the absence of endogenous Areg is not sufficient on its own to alter MuSC expansion (Burzyn et al., 2013).

Although we did not observe changes in MuSC expansion, we next examined the impact of Areg deficiency on other potential cellular targets expressing EGFR, focusing on FAPs. Previous transcriptomic studies have suggested EGFR expression on FAPs (Petrany et al., 2020; Vumbaca et al., 2021). To our knowledge, we are first to show by flow cytometry protein expression of EGFR on FAPs. It was previously reported that there is perivascular fibrosis in muscle from chronic *T. gondii* infected mice (Melchor et al., 2020). We found a muted FAP response in Areg KO mice suggesting that Areg may be promoting this perivascular fibrosis during infection.

Classification of FAPs into subpopulations that carry out unique functions is not well understood. The field of FAPs and identifying distinct FAP populations has largely been moving forward by RNA sequencing assays. One study identified a population of “mesenchymal” stem cells with an inflammatory transcriptional signature that is crucial for immunocyte recruitment in acute injury and dystrophic settings, characterized by upregulation of *Cxcl5*, crucial for neutrophil recruitment (Yaghi et al., 2023). By flow cytometry, two markers, Tie2 and VCAM1, have been used to define FAP subsets (Malecova et al., 2018). Tie2^+^ FAPs are reported to be present in undisturbed muscles but primarily during development in neonatal muscle where Tie2 FAPs are activated (Malecova et al., 2018). This may explain why we do not observe a large population of Tie2^+^ FAPs in adult mice. VCAM1^+^ FAPs are reported as being associated with an inflammatory response in adult life and robustly observed in acute injury settings and suggested to be in a profibrotic state (Malecova et al., 2018). Our study, novel in assaying the dynamics of FAPs during chronic *T. gondii* infection, showed an increase at day 30 of infection but did not display a robust expansion of VCAM1^+^ FAPs as observed during acute injury as previously reported, nor did Areg deficiency impact the dynamics of that subpopulation. This suggests that chronic *T. gondii* infection, while known to cause damage to the muscle, leads to FAP expansion overall but does not impact the expansion of VCAM1^+^ FAP subpopulation.

Another interesting observation we noticed with FAPs during infection is a drastic drop at day 10 of infection, regardless of Areg sufficiency. It has been reported that, after expansion, FAPs undergo apoptosis to return to baseline levels and that macrophages mediate FAP apoptosis via TNFα (Lemos et al., 2015). When macrophages are depleted, VCAM1^+^ FAPs persist (Malecova et al., 2018). Our previously published data show high serum levels of TNFα at 10 days post-infection and day 30 infected muscle has detectable levels of TNFα (Jin et al., 2017; Jin et al., 2018b). Our previous report of an expanded inflammatory macrophage population in combination with the decreased FAP numbers at day 10, supports the idea that these inflammatory macrophages contribute to the acute contraction of FAPs during *T. gondii* infection (Biferali et al., 2019).

A limitation of our study is the unequal biological replicate numbers within the Areg KO cohort. This disparity may have limited the statistical power of certain comparisons. Therefore, the absence of statistically significant differences in some analyses should not necessarily be interpreted as evidence of no biological effect. Despite this limitation, the overall trends observed across independent experiments were consistent and support the conclusions presented here.

Macrophages play an important part in the muscle repair process. While new myofibers arise from MuSCs, their activation and proliferation are influenced by a tightly regulated immune response. At the site of damage, macrophages adopt an inflammatory phenotype that is critical for clearance of cellular debris and is thought to promote subsequent transition to restorative phenotype (Bosurgi et al., 2011; Banerjee et al., 2013). During chronic *T. gondii* infection, we previously reported inefficient macrophage transition resulting in accumulation of inflammatory macrophages in skeletal muscle and unexpected persistent iNOS expression in the restorative compartment (Jin et al., 2017). We also showed that exogenous Areg treatment improved macrophage transition and muscle repair (Jin et al., 2018b). Here we asked whether endogenous Areg is required for macrophage polarization during chronic infection. We find that endogenous Areg deficiency does not impact the presence of either inflammatory or restorative macrophages. It is possible the Effects of exogenous Areg observed previously are due to pharmacologic levels of the cytokine, whereas physiologic Areg levels, even when absent, are insufficient to shift macrophage fate in the context of strong parasite-driven inflammatory cues.

Tregs are critical for macrophage transition during muscle repair and have been reported as an important source of Areg (Villalta et al., 2014; Arpaia et al., 2015). In an inflammatory environment, Tregs can adapt their phenotype to survive, traffic and function (Campbell and Koch, 2011). During *T. gondii*, it was reported that the intestinal Treg compartment diminished and that Tregs acquired a more pathological phenotype, expressing Tbet, a hallmark Th1 transcription factor, as well as producing IFN𝛾 (Oldenhove et al., 2009). In our previous studies we reported that Tregs in the skeletal muscle during chronic *T. gondii* infection expressed higher levels of Tbet and, though not producing IFN𝛾, coincident with inefficient macrophage transition (Jin et al., 2017). Interestingly, Treg depletion in this setting improved macrophage transition and muscle repair, opposite to what is observed in sterile injury models (Burzyn et al., 2013). Therefore, while in acute injury settings, Tregs may be a potentially crucial source of Areg, but during *T. gondii* infection they are tissue injurious. Furthermore, Areg treatment reduced Tbet^+^ Tregs and improved the shift from inflammatory to restorative macrophages, along with better muscle function (Zaiss et al., 2013). Based on these observations, we initially expected that Areg-deficient mice would exhibit increased Tbet^+^ Tregs. Instead, we find that complete loss of Areg results in fewer Tbet^+^ Tregs, phenocopying the effect of exogenous Areg treatment. EGFR expression on Tregs appears to be tissue specific and is not thought to be highly expressed on Tregs in muscle. Therefore, an alternative hypothesis could be that the lower Tbet-expressing Tregs may be a consequence of lower levels of IFNγ from CD8^+^ T cells and not a reflection of a lack of Areg. However, future studies would need to directly test this scenario.

Similarly, when examining CD8^+^ T cells, we did not anticipate an effect on protective immune compartments, because several studies have reported that Areg treatment improves tissue repair without significantly altering pathogen control or systemic immunity (Jin et al., 2018b; Monticelli et al., 2011). CD8^+^ T cells are crucial for control of *T. gondii* infection, with IFNγ production and CD4+ T cells being major factors in protection against the parasite (Suzuki et al., 1989; Suzuki et al., 1990; Tsitsiklis et al., 2019). Furthermore, CD8^+^ T cells are capable of lysing not only infected cells but also the parasite itself (Dotiwala et al., 2016). Given these data, we expected that Areg deficiency would not markedly affect CD8^+^ T cells. Surprisingly, we observed that Areg deficient mice have reduced frequencies and numbers of IFN𝛾 producing CD8^+^ T cells. The mechanism underlying this defect is unclear, as the CD8^+^ T cells do not express EGFR under most conditions, suggesting an indirect effect of Areg (Lozano et al., 2019). It has been reported that Areg-deficient Treg mice have reduced Th1 cells in the brains of *cryptococcus neoformans* infection (Olszewski et al., 2025), which is in agreement with our findings. Interestingly, the decreased presence of IFN𝛾^+^CD8^+^ T cells did not coincide with any changes in parasite burden, showing that the levels of IFN𝛾^+^ produced by both CD4^+^ and CD8^+^ T cells are sufficient for pathogen control and suggesting that IFN𝛾^+^ CD8^+^ T cells may drive the pathogenic program in skeletal muscle Tregs.

Although our study identifies a role for Amphiregulin in shaping FAP and immune responses during chronic *T. gondii* infection, additional experiments will be required to dissect the underlying mechanisms in more detail. *In vitro* assays comparing primary FAPs from WT and Areg KO mice could directly test how Areg influences FAP proliferation and extracellular matrix production. Likewise, profiling cytokine production by muscle macrophages, including regulatory (TGF-*β*, IL-10) and pro-inflammatory (TNF-*α*) mediators, would help clarify how Areg differentially modulates macrophage polarization and tissue repair.

Endogenous Areg act at the intersection of stromal and immune compartments to support muscle regeneration during chronic *T. gondii* infection. In WT mice parasite-induced damage drives expansion and activation of specific FAP subsets (Sca1^+^, CD34^+^Sca1^+^, VCAM1^+^) and leads to the adaptation of Tregs and IFN𝛾^+^CD8^+^ T cells within the inflamed muscle, creating a microenvironment that, together with largely preserved MuSC dynamics, supports efficient repair after CTX injury. In the absence of Areg, FAP expansion is delayed, Tbet^+^ Tregs and IFN𝛾^+^CD8^+^ T cells are reduced, and this altered stromal-immune niche fails to fully sustain regeneration, leading to smaller, more heterogeneous myofibers and increased damaged area after CTX despite similar parasite burden. Notably, Areg-deficient females show a significant reduction in myofiber CSA, whereas males exhibit a comparable shift toward smaller fibers without a change in mean CSA, suggesting that Areg is required to maintain fiber size and architectural integrity in both sexes but that the quantitative impact on CSA is more pronounced in females under chronic infection. Such sex-specific differences are in line with reports that hormonal and intrinsic features of male and female skeletal muscle differentially shape regeneration and remodeling after injury (Velders and Diel, 2013). Furthermore, we did not assess the level of damage in Areg KO mice at earlier time points and therefore cannot rule out the role of Areg early on in response to cardiotoxin injury. Nevertheless, we propose a model that Areg does not primarily control infection, but instead locally controls FAP subset behavior and Treg and CD8^+^ T cell programming to enable effective repair under conditions of chronic muscle damage.

## Data Availability

The original contributions presented in the study are included in the article/Supplementary material, further inquiries can be directed to the corresponding author.
